# Identification and activation of TLR4-mediated signalling pathways by alginate-derived guluronate oligosaccharide in RAW264.7 macrophages

**DOI:** 10.1038/s41598-017-01868-0

**Published:** 2017-05-10

**Authors:** Weishan Fang, Decheng Bi, Ruijin Zheng, Nan Cai, Hong Xu, Rui Zhou, Jun Lu, Min Wan, Xu Xu

**Affiliations:** 1 College of Life Sciences and Oceanography, Shenzhen Key Laboratory of Marine Bioresources and Ecology, Shenzhen University, Shenzhen, 518060 P.R. China; 2 Department of Environmental & Community Medicine, Rutgers University-Robert Wood Johnson Medical School, Piscataway, NJ 08854 USA; 3 School of Science and School of Interprofessional Health Studies, Faculty of Health and Environmental Sciences, and Institute of Biomedical Technology, Auckland University of Technology, Auckland, 1142 New Zealand; 4 Division of Physiological Chemistry 2, Department of Medical Biochemistry and Biophysics, Karolinska Institute, Stockholm, 17177 Sweden

## Abstract

Alginate, a natural acidic polysaccharide extracted from marine brown seaweeds, is composed of different blocks of β-(1, 4)-D-mannuronate (M) and its C-5 epimer α-(1, 4)-L-guluronate (G). Alginate-derived guluronate oligosaccharide (GOS) readily activates macrophages. However, to understand its role in immune responses, further studies are needed to characterize GOS transport and signalling. Our results show that GOS is recognized by and upregulates Toll-like receptor 4 (TLR4) on RAW264.7 macrophages, followed by its endocytosis via TLR4. Increased expression of TLR4 and myeloid differentiation protein 2 (MD2) results in Akt phosphorylation and subsequent activation of both nuclear factor-κB (NF-κB) and mechanistic target of rapamycin (mTOR). Moreover, GOS stimulates mitogen-activated protein kinases (MAPKs); notably, c-Jun N-terminal kinase (JNK) phosphorylation depends on TLR4 initiation. All these events contribute to the production of inflammatory mediators, either together or separately. Our findings also reveal that GOS induces cytoskeleton remodelling in RAW264.7 cells and promotes macrophage proliferation in mice ascites, both of which improve innate immunity. Conclusively, our investigation demonstrates that GOS, which is dependent on TLR4, is taken up by macrophages and stimulates TLR4/Akt/NF-κB, TLR4/Akt/mTOR and MAPK signalling pathways and exerts impressive immuno-stimulatory activity.

## Introduction

Macrophages are important components of the innate immune system, playing a crucial role in host defence against infection through immuno-inflammatory responses, recognition of pathogens and phagocytosis. Indeed, the recognition and phagocytic capacities of macrophages are improved by secretion of pro-inflammatory factors^1^. However, the initial sensing of infection and strengthening of innate immunity are mediated by pattern recognition receptors (PRRs)^2^. As critical PRRs of host defence systems, Toll-like receptors (TLRs) are widely found on the surface of monocytes, macrophages and dendritic cells. TLRs are distinctly required for pathogen recognition by the innate immune system and mediate the production of inflammatory factors and also regulate immune responses^3^. Among TLRs, TLR4 acts as a receptor for lipopolysaccharide (LPS), and associates with the myeloid differentiation protein 2 (MD2) to form a complex to interact with LPS^4^. The formation of TLR4-MD2-LPS complex activates the adaptor of myeloid differentiation factor 88 (MyD88) and drives pro-inflammatory signalling cascades, resulting in the activation of phosphatidylinositol-3-kinase (PI3K) and protein kinase B (Akt or PKB)^5^. It is reported that Akt participates in the nuclear factor-κB (NF-κB) signalling pathway to promote inflammatory responses^6^. The mechanistic target of rapamycin (mTOR) pathway is also critical for signalling downstream of TLR4/Akt, playing roles in cell growth^7^ and immune regulation^8^. It has been demonstrated that both PI3K inhibitors and mTOR inhibitors can reduce LPS-stimulated cytokine secretion in RAW264.7 cells by decreasing Akt phosphorylation^8^. Furthermore, the mitogen-activated protein kinase (MAPK) pathway has a vital function in TLR4 signalling and subsequently the production of pro-inflammatory mediators^3^. Growing evidence suggests that polysaccharides and oligosaccharides from natural sources have potential as immunomodulators, with wide pharmacological applications, by recognizing macrophage cell surface receptors such as TLRs and initiating signal transduction and phagocytosis^9, 10^.

Alginate belongs to a family of natural acidic polysaccharides extracted from marine brown seaweeds. The molecule is composed of different blocks of β-(1, 4)-D-mannuronate (M) and its C-5 epimer α-(1, 4)-L-guluronate (G)^11^, with three types of polymer blocks typically observed: polymannuronate (PM), polyguluronate (PG) and a heteropolymer with random residues of M and G (PMG)^12^. Due to its beneficial bioactivities, alginate has been widely used as a food additive, cosmetic ingredient and pharmaceutical material^13^. With lower molecular weights and viscosities compared with the polymer, alginate oligosaccharide (AOS) appears to possess more physiological activities. For example, AOS prepared by oxidative degradation, which has a carboxyl group at the reducing end, displays anti-inflammatory and neuroprotective effects^14, 15^. Additionally, AOS prepared by enzymatic degradation, which has an unsaturated terminal structure with a C4-C5 double bond, exhibits various immuno-stimulation^16^, anti-tumour^17^, antioxidant and neuroprotective activities^18^. Among these activities, more attention has been paid to the macrophage immuno-inflammatory responses induced by unsaturated guluronate oligosaccharide (GOS) derived from PG. GOS has been found to augment the release of cytokines, such as tumour necrosis factor-α (TNF-α)^16^, and to induce the production of nitric oxide (NO) by activating NF-κB and MAPK signalling pathways^19^ in RAW264.7 cells. GOS also promotes macrophage bactericidal activity by increasing phagocytosis *in vitro* and *in vivo* ^20^. Furthermore, proteomic analysis revealed that GOS improves macrophage homeostasis^21^. However, there is limited knowledge regarding the mechanism by which GOS regulates immune responses in macrophages, which warrants further investigation. The overall goal of the present study is to investigate GOS receptor recognition and transport into cells as well as the mechanisms of GOS-activated immune responses mediated by TLR4-involved signalling in RAW264.7 cells.

## Results

### Determination of endotoxin contamination and macrophage endocytosis of GOS

**Figure 1:**
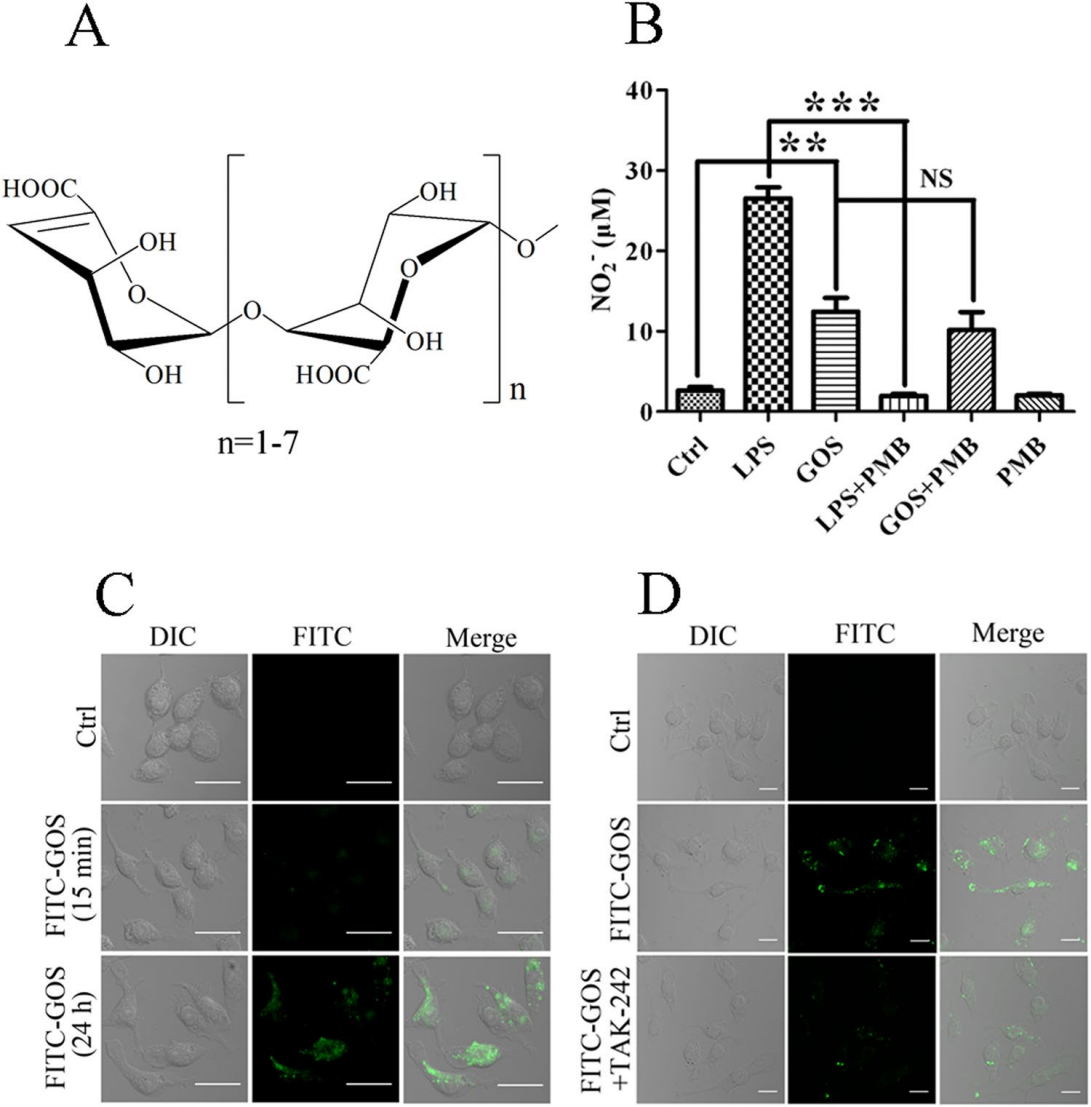
Endotoxin contamination tests and macrophage endocytosis of GOS. (**A**) Stereo-image of structure of alginate-derived GOS. (**B**) RAW264.7 cells were incubated with LPS (1 μg/ml, as a positive control) and GOS (1 mg/ml) in the presence or absence of the LPS inhibitor PMB (0.25 μg/ml) for 24 h. Nitrite levels in the culture medium of treated cells were measured using the Griess assay. (**C**) RAW264.7 cells were incubated with 1 mg/ml FITC-GOS for 15 min and 24 h and observed by laser scanning confocal microscopy (40x). (**D**) RAW264.7 cells were pre-incubated for 2 h with the TLR4 inhibitor TAK-242 (1 μM), then incubated with 1 mg/ml FITC-GOS for 22 h, and observed by laser scanning confocal microscopy (40x). Representative images and results from three independent experiments are shown. Scale bar = 20 μm. NS, no significance. **P < 0.01, ***P < 0.001.

Our earlier studies have shown that GOS increases the production of pro-inflammatory mediators such as NO and TNF-α in RAW264.7 cells^19^. However, endotoxin (LPS) is also an effective inducer of inflammatory mediators. Thus, to eliminate potential false-positive results caused by endotoxin contamination, the GOS used in the current study was purified using an endotoxin-removal column. RAW264.7 macrophages were treated with GOS with or without polymyxin B (PMB), an LPS inhibitor, and NO production was evaluated. As shown in Fig. 1B, NO production by GOS-treated macrophages was not apparently affected by the presence of PMB. In contrast, LPS-stimulated NO production was completely blocked by the addition of PMB, suggesting that the observed GOS-induced macrophage activation was not due to endotoxin contamination.

Next, we labelled GOS with fluorescein isothiocyanate (FITC) and employed confocal laser scanning microscopy to examine whether FITC fluorescence labelled GOS (FITC-GOS) was taken up by macrophages. Weak FITC fluorescence appeared in RAW264.7 cells at 15 min after exposure to FITC-GOS, and fluorescence intensity was markedly enhanced after 24 h. These results indicate that FITC-GOS was taken up by RAW264.7 cells in a time-dependent manner (Fig. 1C). We hypothesized that GOS is recognized by TLR4 on macrophages, therefore, to examine this possibility, we investigated the effect of a TLR4 inhibitor, ethyl (6 R)-6-[N-(2-chloro-4-fluorophenyl) sulfamoyl] cyclohex-1-ene-1-carboxylate (TAK-242), on FITC-GOS endocytosis by RAW264.7 macrophages. As shown in Fig. 1D, the fluorescence intensity was reduced by the addition of TAK-242, indicating that the inhibitor suppressed FITC-GOS endocytosis. However, intracellular FITC-GOS was not completely abolished by TLR4 inhibition. These data reveal that GOS is taken up by macrophages via receptor-mediated endocytosis and that TLR4 is a specific and important contributor but not the only receptor for GOS in this process.

### Effect of GOS on TLR4 expression and TLR4/Akt signalling pathway activation

**Figure 2:**
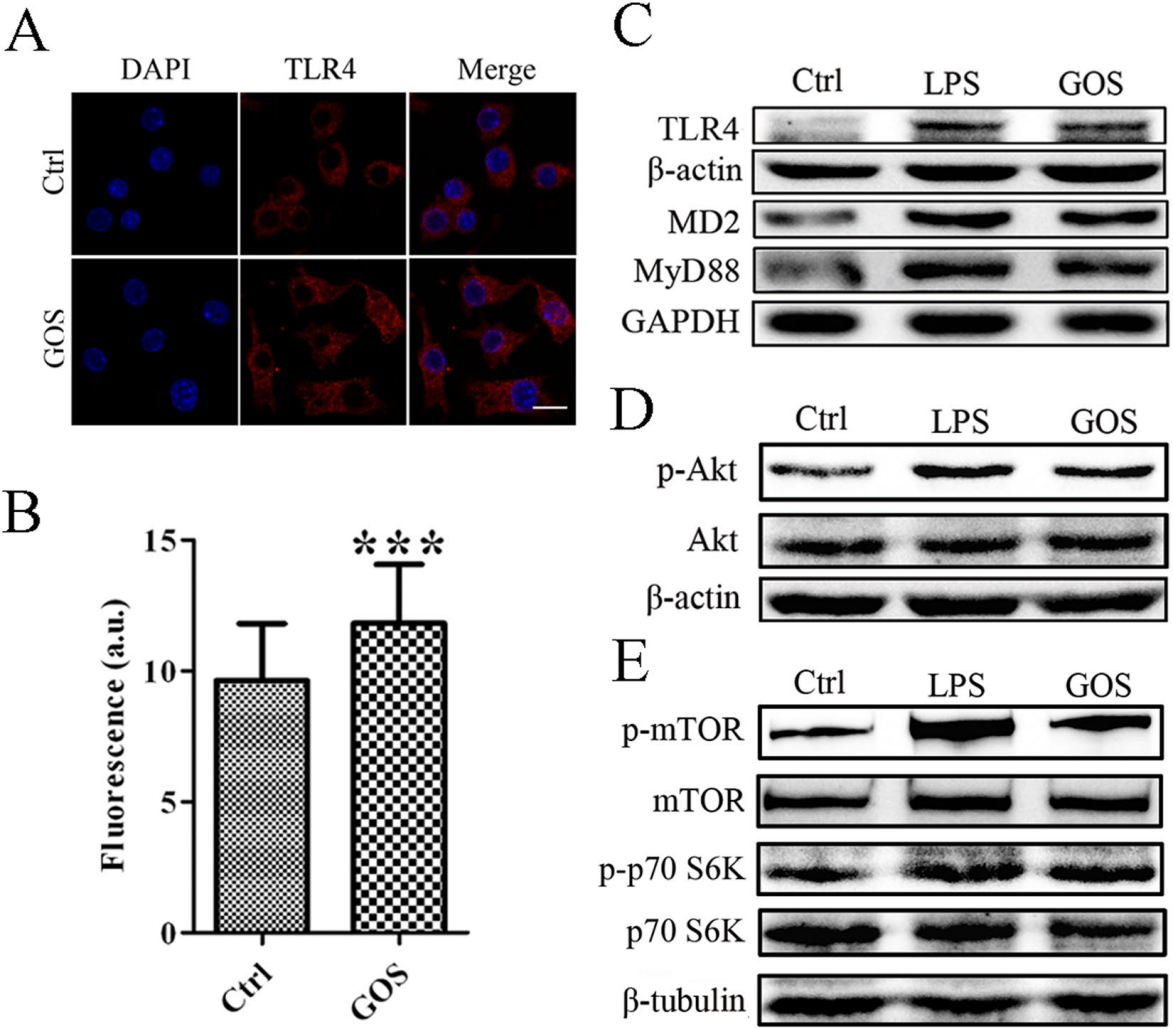
GOS increases TLR4 expression and stimulates Akt/mTOR activation in RAW264.7 cells. (**A**) RAW264.7 cells were treated with 1 mg/ml GOS for 24 h, and TLR4 expression was evaluated by immunofluorescence analysis using an anti-TLR4 antibody. DAPI was used to label nuclei. Images were observed by laser scanning confocal microscopy (40x). (**B**) The average fluorescence intensity of forty cells of each treatment group was measured using ImageJ software. (**C**) RAW264.7 cells were incubated with LPS (1 μg/ml, as a positive control) and GOS (1 mg/ml) for 24 h. The protein expression of TLR4, MD2 and MyD88 was detected by Western blotting. (**D**) Cells were treated with 1 μg/ml LPS (positive control) or 1 mg/ml GOS for 24 h, and phosphorylated and nonphosphorylated Akt were detected by Western blotting. (**E**) Phosphorylation of mTOR and p70 S6K was analyzed by Western blotting. The full-length western blots were presented in supplementary information. Representative images and results from three independent experiments are shown. ***P < 0.001.

TLR4 participates in the transport of GOS into RAW264.7 cells. Accordingly, we further explored the impact of GOS on TLR4 expression and TLR4-mediated signalling. Immunofluorescence staining revealed a notable increase in red fluorescence intensity for the GOS-treated group compared to the blank control group (Fig. 2A), and the intensity was quantified using ImageJ software. As shown in Fig. 2B, GOS-enhanced TLR4 fluorescence was statistically different from that of the control. Consistently, protein expression of TLR4, MD2 and MyD88 was upregulated by GOS treatment, as shown by Western blotting (Fig. 2C). Considering that GOS cannot alter CD14 expression in macrophages, which has been demonstrated by our previous publication^20^, we speculate that GOS could activate TLR4 and MD2, and trigger the MyD88-dependent pathway, leading to the production of pro-inflammatory cytokines without a requirement for CD14.

Our previous findings demonstrated that the upregulation of phosphorylated IκB induced by GOS is associated with NF-κB nuclear translocation^20^, we speculated that Akt phosphorylation contributes to GOS-stimulated signalling. As illustrated in Fig. 2D, the significant increase in Akt phosphorylation detected in GOS-treated samples supports a role for GOS in triggering Akt/NF-κB signalling. Furthermore, it was shown that GOS significantly upregulated the phosphorylation levels of mTOR and p70 S6K, key downstream components of Akt signalling (Fig. 2E). Based on the data shown in Fig. 2D and E, we propose that GOS is a potential elicitor of the Akt/mTOR pathway.

Taken together, GOS upregulates TLR4 surface expression, followed by stimulation of PI3K and subsequent phosphorylation of Akt, resulting in NF-κB translocation into the nucleus and mTOR and p70 S6K activation. We conclude that GOS activates macrophages through upregulation of TLR4, followed by Akt/NF-κB and Akt/mTOR pathway activation.

### Effect of TLR4 and PI3K inhibitors on GOS-activated Akt/NF-κB and Akt/mTOR signalling

**Figure 3:**
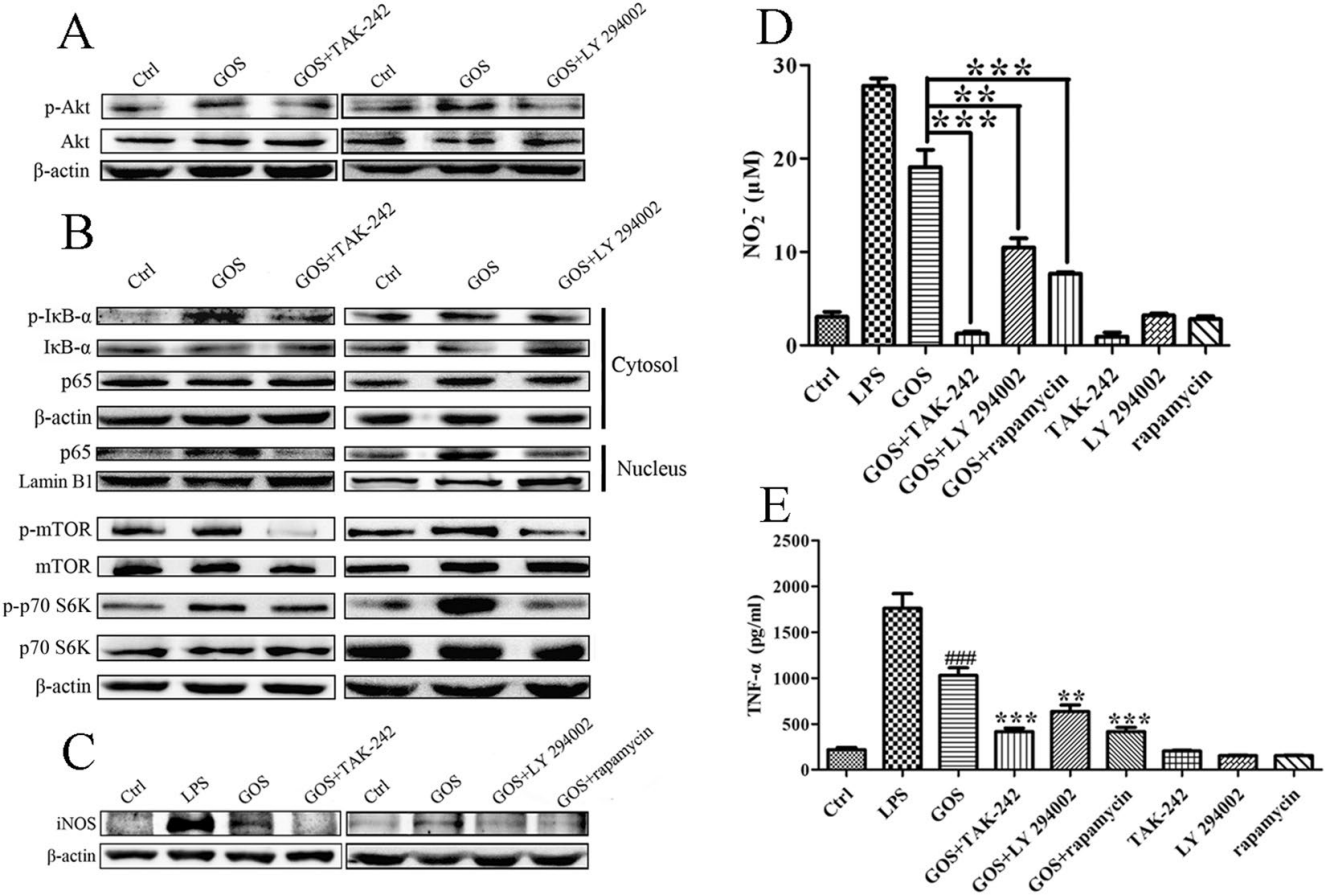
Inhibitors of TLR4 and PI3K suppress GOS-induced activation of Akt/NF-κB and Akt/mTOR pathways. (**A**) RAW264.7 cells were pre-incubated with the TLR4 inhibitor TAK-242 (1 μM) or PI3K inhibitor LY 294002 (10 μM) for 2 h and then co-treated with 1 mg/ml GOS for the indicated time. Akt phosphorylation was analyzed by Western blotting. (**B**) p-IκB, IκB and NF-κB p65 in the cytosolic fraction and p65 in the nuclear fraction were detected by Western blotting. Levels of phosphorylated and nonphosphorylated mTOR and p70 S6K were analyzed by Western blotting. (**C**) RAW264.7 cells were either pre-treated with the TLR4 inhibitor TAK-242 (1 μM), PI3K inhibitor LY 294002 (10 μM) or mTOR inhibitor rapamycin (50 μM) for 2 h prior to GOS treatment or treated with inhibitors alone. iNOS protein expression was detected by Western blotting. The full-length western blots were presented in supplementary information. (**D**) Nitrite production in the culture supernatant of treated cells was measured by the Griess assay. (**E**) TNF-α in the culture supernatant from treated cells was measured by ELISA. LPS (1 μg/ml) was used as a positive control. Representative images and results from three independent experiments are shown. ^#^Indicates significant differences between the control and GOS-treated groups, ^###^ *P* < 0.001. *Indicates significant differences between the GOS-treated and GOS with inhibitor-treated groups, **P < 0.01, ***P < 0.001.

To further determine whether the effects of GOS on the immune system depend on TLR4, PI3K/Akt and TLR4-related pathways, specific inhibitors for TLR4 and PI3K, TAK-242 and LY 294002, respectively, were applied. We found that pre-treatment of RAW264 cells with TAK-242 or LY 294002 suppressed GOS-induced p-Akt expression (Fig. 3A). Both TAK-242 and LY 294002 markedly inhibited GOS-mediated IκB phosphorylation and blocked nuclear translocation of p65 in the Akt/NF-κB pathway as well as phosphorylation of mTOR and p70 S6K in the Akt/mTOR pathway (Fig. 3B). In addition, GOS-induced upregulation of inducible nitric oxide synthase (iNOS) protein expression was reduced by TAK-242, LY 294002 and rapamycin, which are inhibitors of TLR4, PI3K and mTOR, respectively (Fig. 3C). GOS-induced NO and TNF-α production were also effectively arrested by the inhibitors (Fig. 3D and E). Our previous study showed that GOS-induced NO release could be suppressed by the NF-κB inhibitor pyrrolidinedithiocarbamate (PDTC)^20^. Taken together, Akt activation plays a key role in GOS-induced signalling transduction and is closely correlated to downstream NF-κB and mTOR signalling pathways.

### Involvement of TLR4 in GOS-activated MAPK signalling

**Figure 4:**
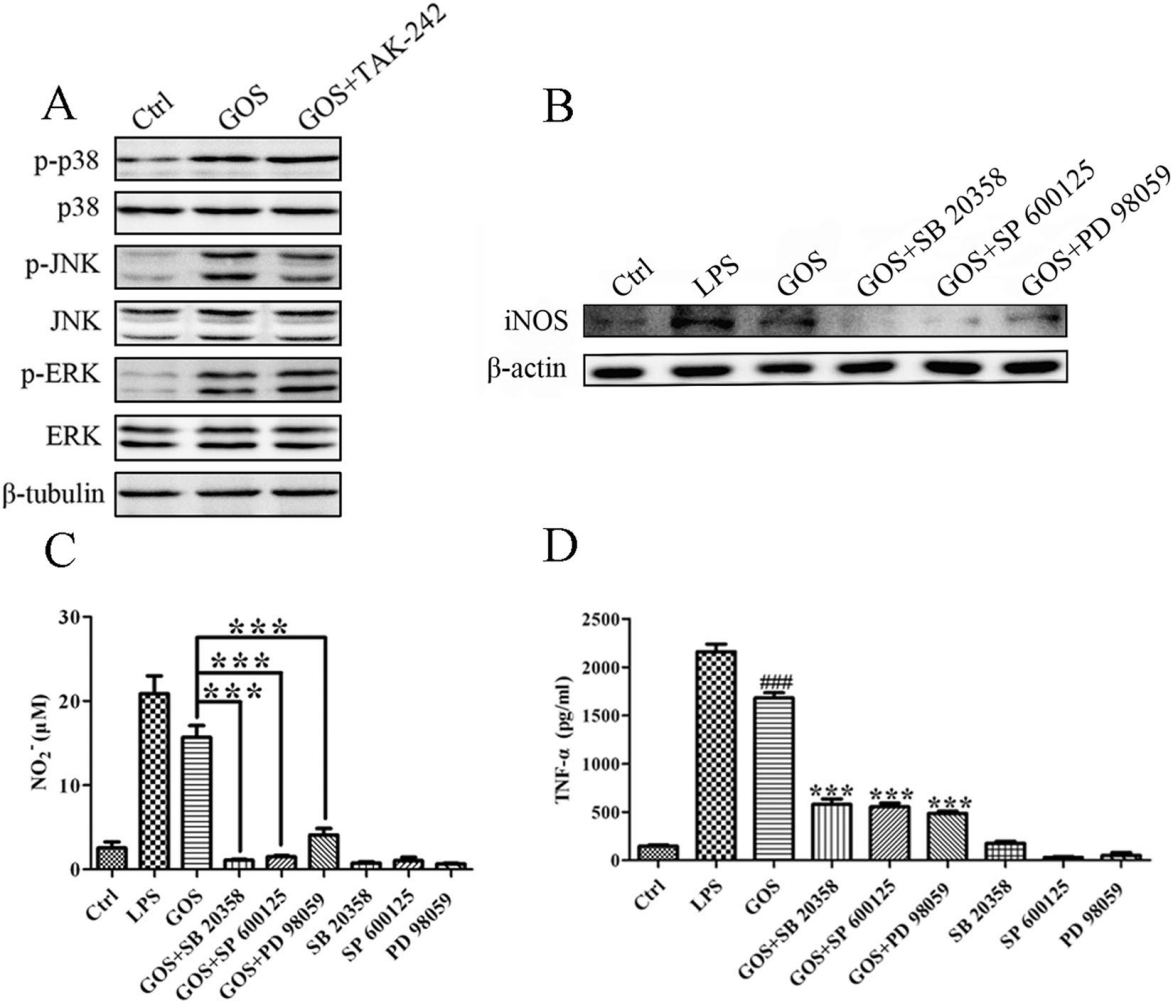
GOS activates the MAPK pathway. (**A**) RAW264.7 cells were pre-incubated with the TLR4 inhibitor TAK-242 (1 μM) and then co-treated with 1 mg/ml GOS for 15 min. Phosphorylated and nonphosphorylated p38, JNK and ERK were detected by Western blotting. (**B**) RAW264.7 cells were either pre-incubated with the p38 inhibitor SB 20358 (10 μM), JNK inhibitor SP 600125 (10 μM) or ERK inhibitor PD 98059 (10 μM) for 2 h prior to GOS treatment or treated with inhibitors alone. iNOS protein expression was assessed by Western blotting. The full-length western blots were presented in supplementary information. (**C**) Nitrite production in the culture supernatant of treated cells was measured by the Griess assay. (**D**) TNF-α in the culture supernatant of treated cells was measured by ELISA. LPS (1 μg/ml) was used as a positive control. Representative images and results from three independent experiments are shown. ^#^Indicates significant differences between the control and GOS-treated groups, ^###^ *P* < 0.001. *Indicates significant differences between the GOS-treated and GOS with inhibitor-treated groups, ***P < 0.001.

In our previous study, GOS was found to possess the ability to activate all MAPKs, including p38, c-Jun N-terminal kinase (JNK) and extracellular signal-regulated kinase (ERK)^19^. However, it remains unknown whether the phosphorylation of all MAPKs by GOS is mediated by TLR4 and related to the production of inflammatory factors. Therefore, we utilized TAK-242 to examine the role of TLR4 in GOS-induced p38, JNK and ERK phosphorylation. As shown in Fig. 4A, the expression of only p-JNK was partly suppressed by TAK-242, whereas phosphorylation of p38 and ERK was not affected. Inhibitors of p38 (SB 20358), JNK (SP 600125) and ERK (PD 98059) were then applied to investigate the contribution of MAPKs in GOS-stimulated inflammatory mediator production. We determined that GOS-induced iNOS expression (Fig. 4B), NO release (Fig. 4C) and TNF-α secretion (Fig. 4D) were all significantly suppressed by the three inhibitors. These data reveal that GOS increases phosphorylation of p38, JNK and ERK MAPKs, resulting in the production of NO and TNF-α. However, the phosphorylation of only JNK depends on TLR4 initiation after GOS recognition, and p38 and ERK are not mediated by TLR4.

### Knockdown of TLR4 in RAW264.7 cells suppresses GOS-induced activation of signalling

**Figure 5:**
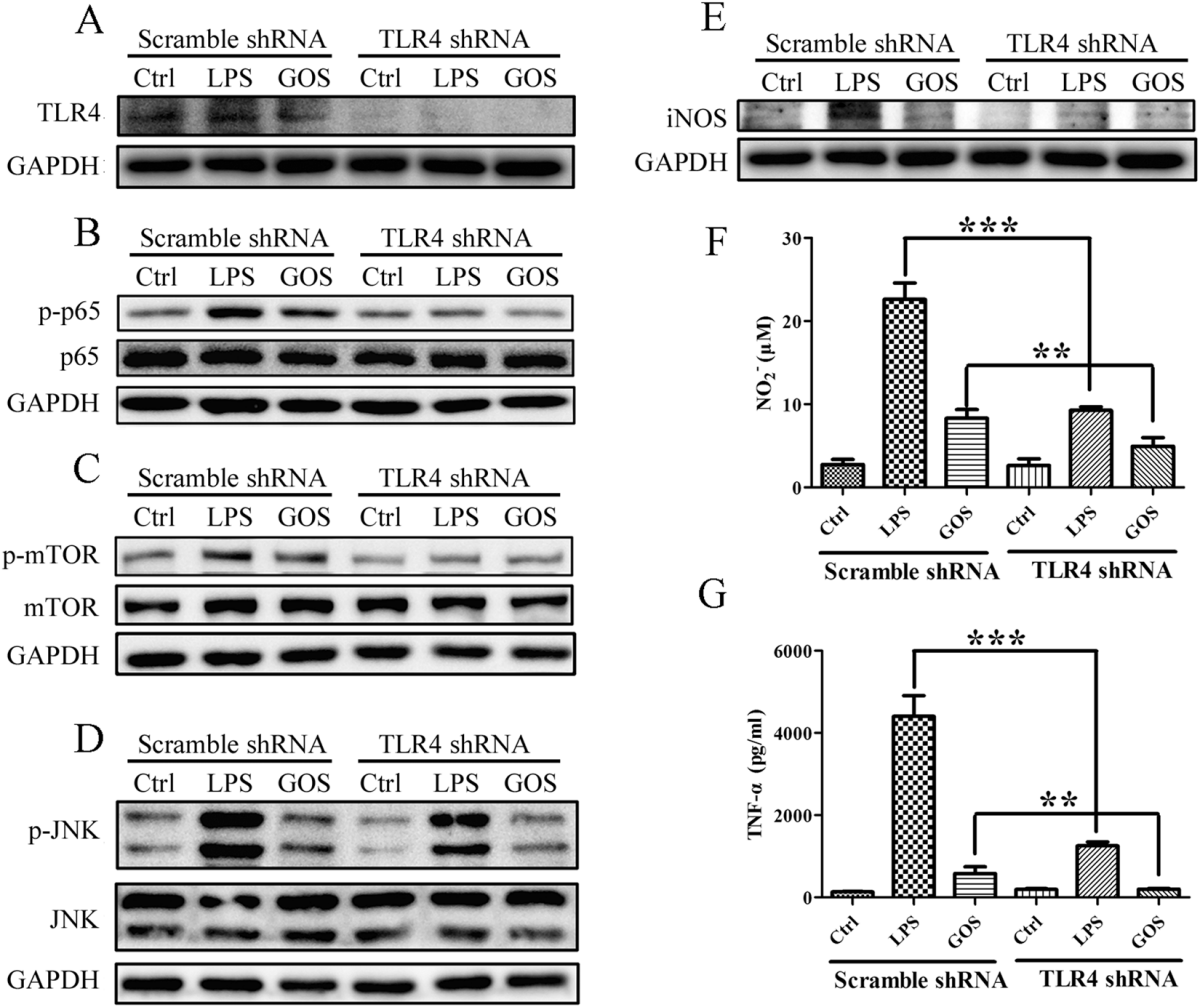
Knockdown of TLR4 with shRNA suppresses GOS-induced activation of signal transduction. (**A**) RAW264.7 cells were infected with lentivirus expressing either scramble shRNA or TLR4 shRNA. After treatment of LPS (1 μg/ml) (as a positive control) or GOS (1 mg/ml), TLR4 protein expression was detected by Western blotting. (**B**–**D**) Phosphorylated and nonphosphorylated p65 (**B**), mTOR (**C**) and JNK (**D**) were detected by Western blotting. (**E**) iNOS protein expression was assessed by Western blotting. The full-length western blots were presented in supplementary information. (**F**) Nitrite production in the culture supernatant of treated infected cells was measured by the Griess assay. (**G**) TNF-α in the culture supernatant of treated infected cells was measured by ELISA. Representative images and results from three independent experiments are shown, **P < 0.01, ***P < 0.001.

To further confirm the role of TLR4 in RAW264.7 cells, we infected RAW264.7 cells with either lentivirus containing TLR4 shRNA or scramble shRNA. Compared with LPS- (positive control) or GOS-treated groups in the scramble shRNA, TLR4 protein expressions in LPS- or GOS-treated groups of the TLR4 shRNA were significantly decreased, respectively (Fig. 5A). The p-p65 and p-mTOR protein expressions induced by LPS (positive control) or GOS were increased in the scramble shRNA group and were inhibited in TLR4 knockdown cells (TLR4 shRNA) (Fig. 5B and C). A similar trend was observed for p-JNK protein expression in RAW264.7 cells transfected with TLR4 shRNA (Fig. 5D). In addition, increases in LPS-induced or GOS-induced pro-inflammatory mediators such as iNOS (Fig. 5E), NO (Fig. 5F) and TNF-α (Fig. 5G) were partially inhibited with TLR4 gene knockdown. These data indicate that TLR4 plays a vital regulatory role in the macrophage activation induced by GOS. We speculate that GOS triggers immune response in macrophages depending on TLR4 and may interact with it.

### Effect of GOS on RAW264.7 cell cytoskeleton organization

**Figure 6:**
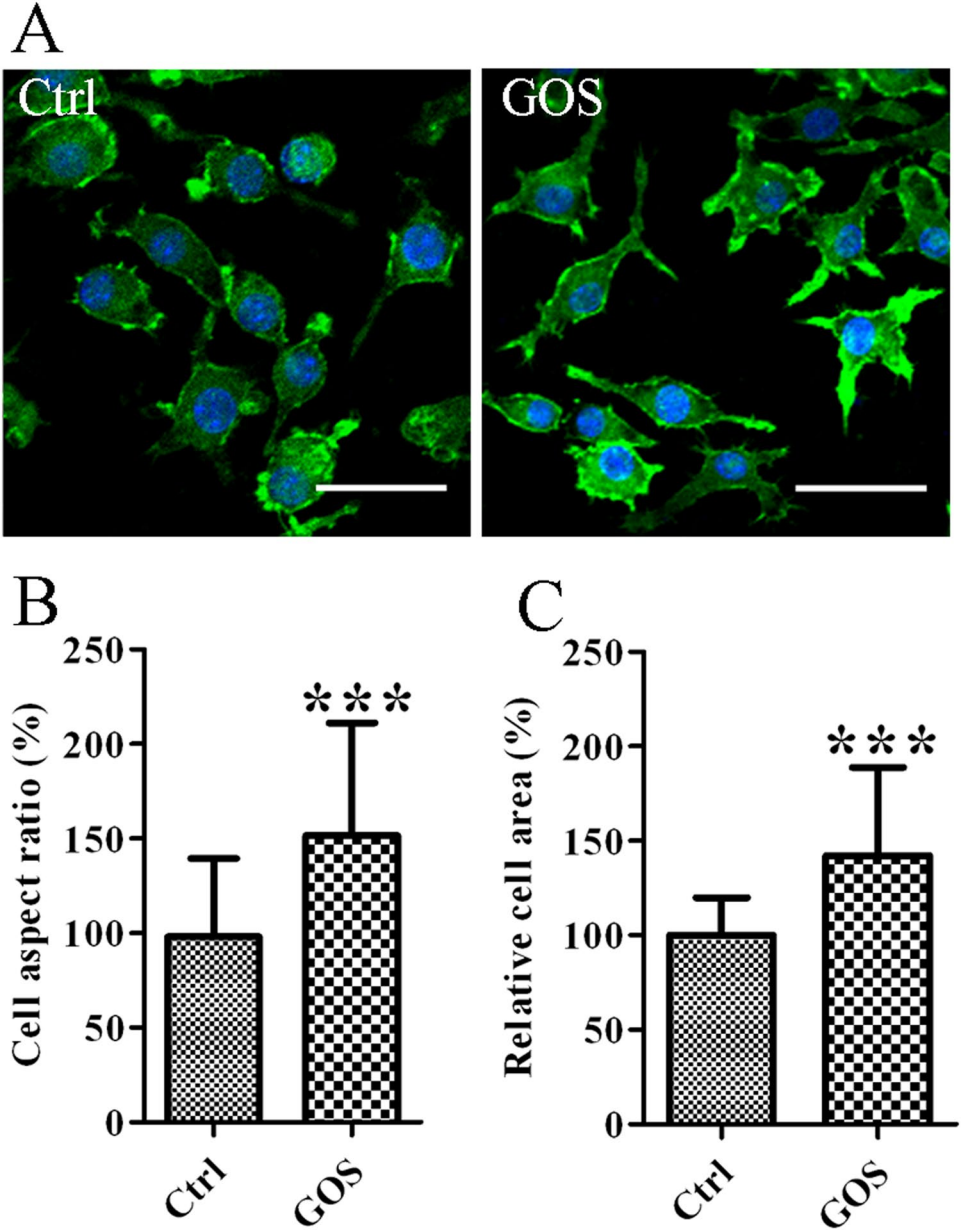
GOS induces changes in the morphology and cytoskeleton of RAW264.7 cells. (**A**) RAW264.7 cells were treated with 1 mg/ml GOS or medium alone (Ctrl) for 24 h and stained with FITC-phalloidin to visualize F-actin. Cell morphology was observed by laser scanning confocal microscopy (40x). Scale bar = 20 μm. GOS-induced morphological changes including the cell aspect ratio (**B**) and cell area (**C**) were quantitatively analyzed using ImageJ software. ***P < 0.001.

mTOR pathway activation is involved in cytoskeleton remodelling and cell migration^22^. Thus, we hypothesized that GOS has the ability to remodel the cytoskeleton in RAW264.7 macrophages because it activates the Akt/mTOR pathway. We observed RAW264.7 cells treated with GOS to be irregular or spindle-like in shape, with more extended pseudopodia (Fig. 6A). These changes in cell shape were quantified as cell aspect ratios (long side:short side) and the cell surface area using ImageJ software. Compared with blank control cells, GOS-treated cells showed significant increases in both cell aspect ratios and relative cell area (Fig. 6B and C).

### Effect of GOS on macrophage proliferation in mouse peritoneum

**Figure 7:**
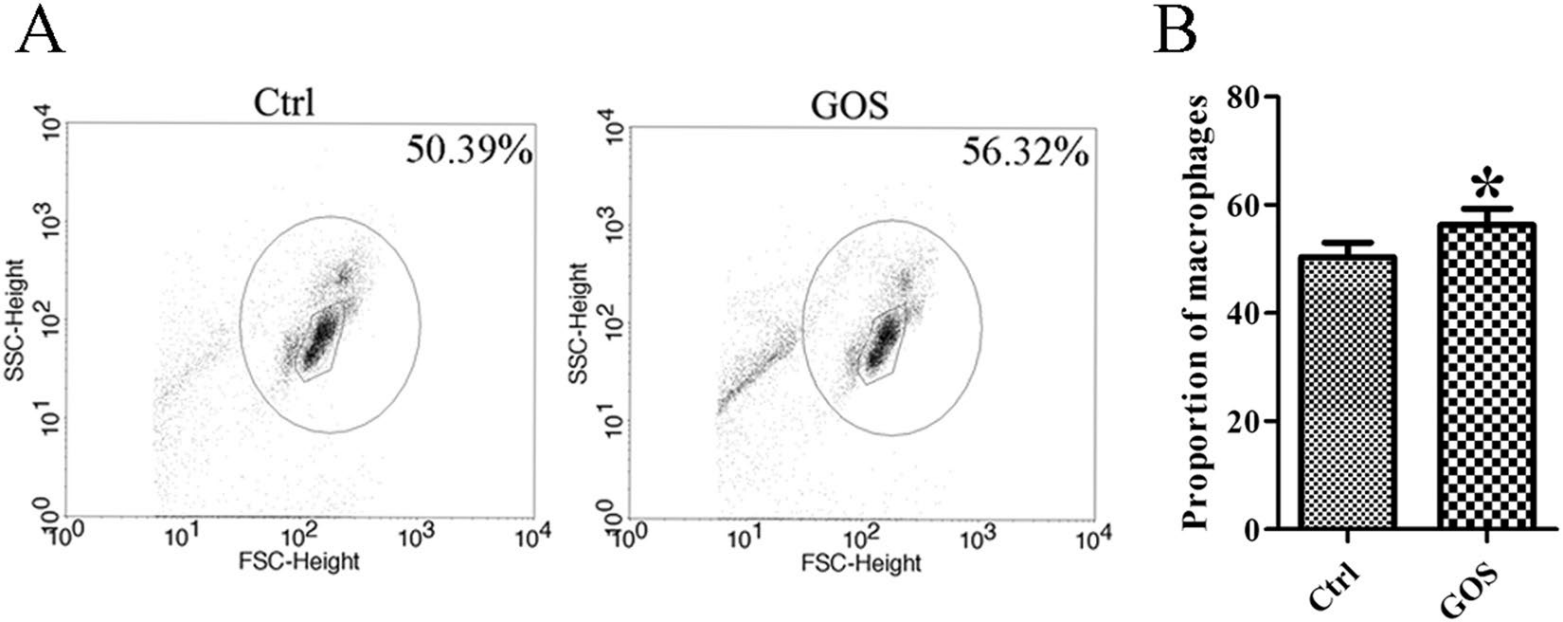
GOS induces macrophage proliferation in mice ascites. (**A**) Male BALB/c mice received an *i.p*. injection of 100 μl PBS or GOS (2 mg/mouse) for 24 h. Peritoneal fluid cells were collected and analyzed by FACS. (**B**) Data are expressed as the percentage of total cells and represent the means ± SD (*n* = 5). *P < 0.05.

Cell growth is closely correlated with cell proliferation in various organs, tissues, and even tumours, and is considered to be an mTOR-dependent process that is extremely sensitive to the mTOR inhibitor rapamycin^7^. To determine the effect of GOS on macrophage proliferation, we treated male BALB/c mice with GOS via *i.p*. injection, collected primary macrophages from ascites, and determined the number (proportion) of macrophages using FACS. A pronounced increase in macrophage proportions was detected in GOS-treated mice compared to the control (Fig. 7A), and statistical analysis of the fluorescence intensity showed a trend that was consistent with the flow cytometric plots (Fig. 7B). Therefore, we suggest that the increased number of peritoneal macrophages could effectively improve non-specific immunity, thereby boosting pathogen removal efficiency in mice.

**Figure 8:**
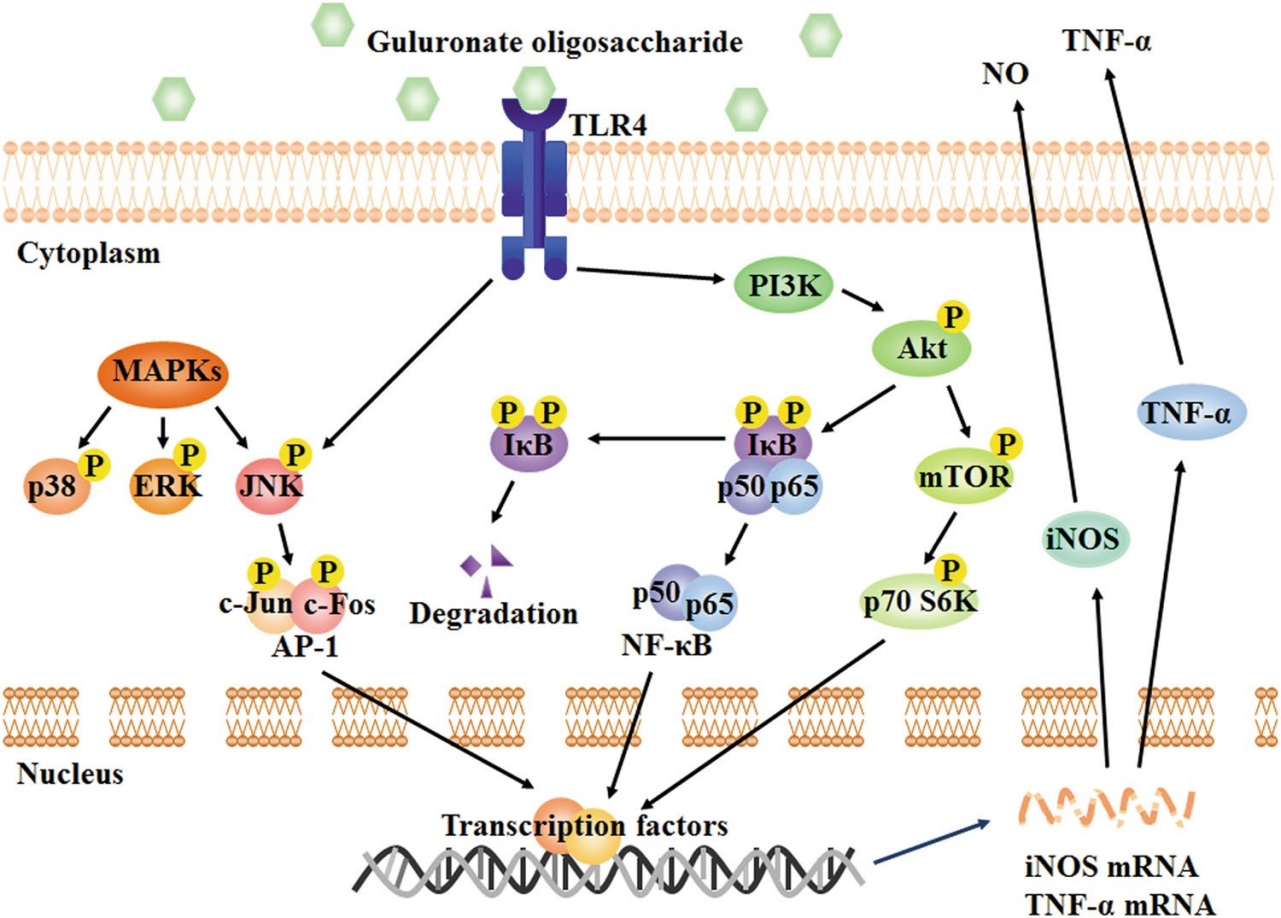
Signalling pathways involved in the macrophage activation effect of alginate-derived guluronate oligosaccharide.

In summary, our findings demonstrate that GOS can be taken up by RAW264.7 cells via endocytosis through TLR4. GOS stimulates macrophage activation via TLR4-related Akt/NF-κB, Akt/mTOR and MAPK pathways, followed by the production of NO and TNF-α, as illustrated in Fig. 8. Furthermore, GOS induces cytoskeleton remodelling to increase the cell aspect ratio and surface area. Additionally, GOS can also increase macrophage proliferation in mice ascites.

## Discussion

Endocytosis, a characteristic of macrophages, includes phagocytosis of bacteria or other large particles, pinocytosis of colloids, and receptor-mediated endocytosis^23^. Multiple receptors, such as TLRs, Fcγ receptor (FcγR) and scavenger receptor (SR), have been found to activate signalling pathways and to be involved in endocytosis^24^. It was indicated that FITC-GOS was taken up by RAW264.7 cells (Fig. 1C). Our previous results demonstrated that pre-treatment with GOS could reduce LPS-stimulated cell morphological changes and damage^21^, suggesting that GOS is a potential competitive antagonist of macrophage cell surface receptors. Additionally, GOS has been found to activate NF-κB and MAPKs^19^, both of which are downstream of TLR4. Therefore, we hypothesized that GOS interferes with LPS via competitive interaction to macrophage cell surface receptors, such as TLR4, a well-known LPS-recognizing receptor. To examine this possibility, we selected TAK-242 which was reported to completely suppress the production of cytokine and nitric oxide (NO) induced by TLR4 ligand, as a TLR4 inhibitor, and investigated its effect on FITC-GOS endocytosis by RAW264.7 macrophages. As expected, we verified that GOS was taken up by macrophages via receptor-mediated endocytosis and GOS endocytosis was suppressed partially by TAK-242, indicating that TLR4 was a specific and important contributor but not the only receptor for GOS in this process (Fig. 1D). Previous studies have shown that GOS stimulated FcγRI and FcγRII expression on the macrophage surface, resulting in increased phagocytosis capacity^20^. Moreover, TLR2 activation by GOS was also evident in RAW264.7 cells^16^. Further investigations are needed to discover more macrophage cell surface receptors to GOS.

Exogenous antigens such as polysaccharide enter cells by recognizing cell surface receptors, binding to which then activates downstream intracellular signalling to regulate immune responses^9^. As a critical member of the TLR family, TLR4 has functions in activating innate immune cells and triggering inflammatory signal transduction^25^. We also demonstrated that GOS enhanced TLR4 expression (Fig. 2A–C) and the inflammatory signal transduction triggered by GOS was partly suppressed in RAW264.7 cells with TLR4 gene knockdown (Fig. 5), suggesting that GOS may recognize and interact with TLR4.As reported by Nam *et al*., Akt phosphorylation activates IκB kinase (IKK), and the inhibitory protein (IκB) of NF-κB is then degraded by phosphorylated IKK through the ubiquitin proteasome pathway, resulting in the release of NF-κB and its translocation from the cytoplasm to the nucleus. NF-κB then initiates target gene expression and induces the production of inflammatory mediators including iNOS, NO and pro-inflammatory cytokines such as TNF-α, interleukin-1 (IL-1), and IL-6^26^. Combined with our previous findings that the upregulation of phosphorylated IκB induced by GOS is associated with NF-κB nuclear translocation^20^, we speculated that Akt phosphorylation contributes to GOS-stimulated signalling. In addition, the Akt/mTOR signalling pathway is reported to participate in various processes, including cell growth, autophagy, apoptosis and immune regulation^27^, and activated Akt was found to induce expression of IL-6, TNF-α and IL-12 by initiating phosphorylation of mTOR and p70 S6K^28^. According to current findings (Figs 2D,E and 3), we propose that GOS-mediated immune regulation in RAW264.7 macrophages is divided into the following major steps: (1) GOS the surface receptor TLR4; (2) PI3K is activated by TLR4 and subsequently induces Akt phosphorylation; (3) phosphorylated Akt triggers IκB phosphorylation, resulting in (4) the release and translocation of NF-κB into the nucleus; (5) mTOR and p70 S6K are also activated by p-Akt; (6) all these factors work together or separately to induce the production of inflammatory mediators.

The MAPK pathway is one of the most important pathways downstream of TLR4, and it also contributes to immune responses by regulating the levels of TNF-α, IL-1β, IL-6, iNOS and NO^29^. An increasing number of studies in recent years have proven that various oligosaccharides, including chitosan oligosaccharide, alginate oligosaccharide and rice bran feruloylated oligosaccharide, regulate immune functions via the MAPK pathway^30–32^. Our previous results showed that GOS could activate all MAPKs, including p38, JNK and ERK^19^. In this study, we further conclude that GOS increases phosphorylation of MAPKs, followed by activation of downstream transcription factors; as a consequence, gene transcription is initiated, which results in the production of NO and TNF-α. However, the phosphorylation of only JNK depends on TLR4 initiation after GOS recognition, and p38 and ERK are not mediated by TLR4. It is known that various receptors, including TLR2, SR, mannose receptor (MR), complement receptor (CR) and dectin-1, are also associated with the biological activities of polysaccharides^33, 34^. Our previous studies also show that GOS can enhance the phagocytic function on macrophages by upregulating FcγII and FcγIII expression^20^. Therefore, we speculate that other GOS-recognizing receptors exist. Notably, TAK-242 can inhibit the LPS-induced phosphorylation of p38, JNK and ERK in RAW264.7 cells^35^, while TAK-242 suppresses the GOS-induced phosphorylation of only JNK, but not p38 and ERK (Fig. 4A). Our results here suggest that although GOS shares TLR4 with LPS in macrophage cell surface, their output signals are different.

mTOR pathway activation is involved in cytoskeleton remodelling and cell migration, whereby mTOR regulates the phosphorylation of the actin-remodelling proteins and downstream effector p70 S6K^22^. Pseudopod elongation, irregular shape and increased surface area have been described as common morphological changes induced in RAW264.7 cells by GOS or LPS. The observed changes in macrophage morphology caused by LPS were accompanied by distinct cytotoxicity characterized by loss of membrane integrity, a prominent nucleus and chromatin condensation^21, 36^. In contrast, the GOS-treated cells retained a healthy status, and GOS treatment reduced and repaired the morphological damage caused by LPS^21^. In addition, we have reported that GOS promotes macrophage phagocytosis^20^. It appears that the increases in relative cell area and pseudopodia found in the current study can be beneficial for enhancing macrophage contacts with surrounding pathogens, which in turn, promotes their phagocytosis.

LPS as a component of the Gram-negative bacteria outer membrane, is the principal stimulator of the innate immune system^37^. TLR4 acts as a specific pattern-recognition receptor for LPS, and each of the target proteins involved in the TLR4 signalling pathway activated by LPS has been clarified clearly in the field of innate immune^3^. We used LPS as the positive control of this study. The influences of respectively LPS and GOS on signalling transduction in macrophages are different. Although LPS is recognized by TLR4, but they do not make contact directly. Firstly, LPS is recognized by lipopolysaccharide binding protein (LPB) and transported to the surface of macrophages with the help of LPB and CD14. MD2 and CD14 form a complex to interact with TLR4^38^. Then, the formation of TLR4-MD2-CD14-LPS complex activates MyD88 and drives pro-inflammatory signalling cascades, resulting in the activation of PI3K and Akt^5^. It has been reported that LPS can increase the expression of TLR4, MD2, CD14 and MyD88^39, 40^. We also found that GOS enhanced the expression of TLR4, MD2 and MyD88 (Fig. 2C), but it did not alter the expression of CD14^20^. It is indicated that both LPS and GOS can stimulate TLR4 and MD2, and trigger the MyD88-dependent pathway, subsequently inducing the production of pro-inflammatory cytokines. CD14 is required for above process activated by LPS, but it is not required for that by GOS. MD2 is normally regarded as TLR4 co-receptor molecule^41^. The expression of MD2 was upregulated by GOS, but the function of MD2 in signalling transduction induced by GOS is still unknown.

In this study, we come to a conclusion that GOS stimulates the surface receptor TLR4 and its downstream PI3K/Akt/NF-κB and PI3K/Akt/mTOR signalling pathways. Mounting evidences show that LPS also induces above signalling transduction process via recognition of TLR4^3^. Both LPS and GOS can increase phosphorylation of MAPKs. All these factors work together or separately to induce the production of inflammatory mediators triggered by LPS and GOS. Although GOS shares TLR4 with LPS in macrophage cell surface, their output signals are a little bit different. For example, the phosphorylation of only JNK depends on TLR4 initiation after GOS treatment, and p38 and ERK are not mediated by TLR4 (Fig. 4A), while the phosphorylations of p38, JNK and ERK increased by LPS are all dependent on TLR4^35^. Combined with previous reports by our group^20^ and other authors^16^, other GOS-recognizing receptors exist, including FcγII, FcγIII and TLR2. Other potential receptors are necessary to be investigated to clarify the whole mechanism of GOS-induced immune response in macrophages in the further study.

Our study provides convincing evidence of the beneficial effects of GOS on the activation of macrophages via TLR4-mediated signalling. We believe that our study is the first to investigate that TLR4 serves as a specific and decisive contributor to GOS endocytosis into macrophages. Considering that GOS is derived from an abundant natural source, GOS is a promising food or medicine additive to potentially serve as an immune enhancer with unique immunomodulatory capabilities.

## Methods

### Materials

Alginate, LPS, PMB, FITC-phalloidin, FITC-LPS and 4′,6-diamidino-2-phenylindole (DAPI) were obtained from Sigma-Aldrich (St. Louis, MO, USA). RPMI-1640 medium, Dulbecco’s modified Eagle’s medium (DMEM), penicillin and streptomycin were purchased from Hyclone (Logan, UT, USA). Foetal bovine serum (FBS) was obtained from Biological Industries (Beit-Haemek, Israel). Anti-mouse TLR4, MD2, MyD88, F4/80 and Ly6G antibodies were from purchased Abcam (Cambridge, UK). Antibodies against Akt, phosphor-Akt (p-Akt), NF-κB p65, IκB, phosphor-IκB (p-IκB), mTOR, phosphor-mTOR (p-mTOR), p70 S6 kinase (p70 S6K), phosphor-p70 S6K (p-p70 S6K), p38, phosphor-p38 (p-p38), JNK, phosphor-JNK (p-JNK), ERK, phosphor-ERK (p-ERK) and iNOS were purchased from Cell Signaling Technology (Beverly, MA, USA). Anti-β-actin, -β-tubulin, -GAPDH and -Lamin B1 antibodies were purchased from Proteintech (Hubei, China). Inhibitors LY 294002, SB 20358, SP 600125 and PD 98059 were obtained from Selleck (Shanghai, China) and TAK-242 and rapamycin from Invitrogen (Carlsbad, CA, USA). The other chemicals were all purchased from Macklin Biochemical Technology (Shanghai, China).

### Preparation of GOS and FITC-GOS

GOS was depolymerized from PG using alginate lyase, as described previously^20^. The GOS molecular weight and degree of polymerization were determined by electrospray ionization mass spectrometry (ESI-MS). GOS was found to be composed of α-L-guluronate dimer to octamer (G2-G8)^21^ (Fig. 1A). To reduce endotoxin levels below the limit of detection, the GOS used in the present study was pre-treated with Detoxi-Gel^TM^ Endotoxin Removing Columns (Thermo Scientific, Hudson, NH, USA).

For endocytosis studies, GOS was conjugated to FITC. GOS was dissolved in 2 M ethylenediamine in 10% acetic acid and mixed with 4 M sodium cyanoborohydride at 45 °C overnight in the dark. After dialyzing against distilled water, the dialysate was mixed with 10 mg/ml FITC-methanol solution for 24 h in the dark. The FITC-conjugated oligosaccharide was precipitated from the liquid using excess 95% ethanol, centrifuged at 3,500 rpm for 10 min, re-suspended, dialyzed, and freeze-dried. FITC-GOS was examined using thin layer chromatography (TLC) aluminium sheets (Silica Gel 60; Merck, Germany), and FITC-GOS fluorescence was confirmed using a camera obscura ultraviolet analyzer (Yuhua Instruments Equipment Co., Ltd., Henan, China).

### Cell culture

Murine macrophage RAW264.7 cells were maintained in RPMI-1640 medium; human embryonic kidney (HEK293FT) cells were cultured in DMEM. The media were supplemented with 10% FBS, 100 μg/ml streptomycin and 100 IU/ml penicillin, and the cells were grown in an incubator at 37 °C with a humidified 5% CO_2_ atmosphere.

### Lentivirus construction and infection

Scramble short-hairpin RNAs (shRNA) and shRNAs targeting nucleotides 1246–1264 of the mouse TLR4 mRNA were annealed and cloned into the GV248 lentiviral vector. The sequences were as follows: scramble shRNA, 5′-TTCTCCGAACGTGTCACGT-3′; TLR4 shRNA, 5′-CATCATTATGAGTGCCAAT-3′. HEK293FT cells were infected with the TLR4 shRNA or scramble shRNA vector along with viral packaging plasmids (pLP1, pLP2 and pLP-VSVG) using Lipofectamine 3000 (Invitrogen, CA, USA) to produce retroviral particles. The retroviral particles were collected and purified on days 2 and 3 after infection. RAW264.7 cells were then infected with the retroviruses containing TLR4 shRNA or scramble shRNA in the presence of polybrene. Puromycin-resistant clones were selected, and TLR4 protein expression levels were detected by Western blot analysis.

### Measurement of NO production

Adherent RAW264.7 cells in 96-well plates (2 × 10^5^ cells/well) were pre-treated with vehicle or various concentrations of individual inhibitors for 2 h in serum-free RPMI-1640 medium, followed by co-culture with 1 μg/ml LPS (positive control) or 1 mg/ml GOS for an additional 22 h. Next, 50 μl of culture supernatant from each well was mixed with 100 μl of Griess reagent [1% sulfanilamide, 0.1% *N*-(1-naphthyl)-ethylenediamine dihydrochloride, and 2.5% phosphoric acid] and incubated at room temperature (RT) for 5 min. Sodium nitrite was used to construct a standard curve. Nitrite production was evaluated by measuring absorbance at 540 nm using a Spectra Max microplate reader (Thermo Scientific, Hudson, NH, USA).

### Western blot analysis

After various treatments, cells were washed with phosphate-buffered saline (PBS) and lysed with RIPA buffer (Beyotime Institute Biotech, Jiangsu, China) on ice for 25 min. The supernatant was collected after centrifuging at 12,000 × *g* at 4 °C for 30 min, and nuclear and cytoplasmic fractions were separated using a nuclear extraction kit according to the manufacturer’s protocol. The protein content was assessed using the BCA assay (Auragene Biosciences, Hunan, China). Protein (30 μg) for each sample was loaded, separated by 8% or 12% sodium dodecyl sulfonate-polyacrylamide gel electrophoresis (SDS-PAGE) and transferred to polyvinylidene fluoride (PVDF) membranes. The membranes were blocked with 5% skim milk at RT for 2 h and incubated overnight with primary antibodies (1:1000) at 4 °C. After washing three times with TBS containing 0.5% Tween 20 (TBST), the membranes were treated with the corresponding secondary antibody at 37 °C for 2 h. After washing three times with TBST, densitometric analysis of each protein was carried out using a chemiluminescence (ECL) kit (Thermo Scientific, Hudson, NH, USA).

### Measurement of TNF-α secretion

Adherent RAW264.7 cells in 96-well plates (2 × 10^5^ cells/well) were pre-treated with vehicle or various concentrations of individual inhibitors for 2 h in serum-free RPMI-1640 medium, followed by co-culture with 1 mg/ml GOS for an additional 22 h. The culture supernatants were collected, and the levels of TNF-α were measured using an ELISA kit (Neobioscience Technology Company, Guangdong, China) following the manufacturer’s instructions.

### Determination of macrophage endocytosis of FITC-GOS

RAW264.7 cells (6 × 10^5^ cells/well) were seeded onto 6-well chamber slides. After 4 h, the cells were treated with medium alone or 1 mg/ml FITC-GOS for 15 min or 24 h. To evaluate the role of TLR4 in GOS endocytosis, the cells were pre-treated with 1 μM TLR4 inhibitor (TAK-242) for 2 h and then co-incubated with FITC-GOS for 22 h. Next, the cells were fixed with 4% paraformaldehyde in PBS and observed using laser scanning confocal microscopy (Carl Zeiss Jena Gmbh, Jena, Germany).

### Immunofluorescence analysis

RAW264.7 cells (6 × 10^5^ cells/well) were cultured on 6-well chamber slides and treated with PBS, 1 μg/ml FITC-LPS (positive control) or 1 mg/ml FITC-GOS for 24 h. The cells were fixed with 4% paraformaldehyde in PBS at RT. After washing with PBS, the cells were permeabilized with 0.2% Triton X-100 in PBS at RT for 30 min. After 60 min of blocking with 1% (w/v) goat serum in PBS at 37 °C, the cells were incubated with an anti-TLR4 antibody diluted in PBS (1:200) on ice overnight, washed three times with ice-cold PBS, and stained with an Alexa Fluor 488-conjugated secondary anti-mouse antibody at 37 °C for 2 h. Nuclei were counterstained with DAPI for 15 min. Fluorophores were visualized using laser scanning confocal microscopy, and the images were analyzed using ImageJ software (National Institutes of Health, Bethesda, MD, USA).

### Morphological analysis

RAW264.7 cells (6 × 10^5^ cells/well) on 6-well chamber slides were treated with medium alone or 1 mg/ml GOS for 24 h. The cells were fixed with 4% paraformaldehyde and mixed with 0.2% Triton X-100 for 30 min at RT. After washing three times with PBS, the cells were stained with FITC-phalloidin for 1 h, followed by DAPI staining for 10 min. Morphological changes were monitored by laser scanning confocal microscopy, and the images were analyzed using ImageJ software.

### *In vivo* determination of macrophage proliferation

Male seven-week-old BALB/c mice were purchased from Guangdong Laboratory Animals Monitoring Institute (Guangzhou, Guangdong, China) and fed in a specific pathogen free (SPF) environment. The animal experiments were approved by the Institute of Marine Biotechnology at Shenzhen University according with relevant guidelines and regulations. The mice were received an intraperitoneal (i.p.) injection of 100 μl PBS or GOS (2 mg/mouse). After 24 h, the mice were killed, and the peritoneal cavity was lavaged with 500 μl sterile PBS containing 5 μM EDTA. The lavage fluid was collected, and the cells were washed and re-suspended in 100 μl PBS containing 1% BSA. Next, the cells (1 × 10^6^) were treated with fluorescently conjugated anti-F4/80 and anti-Ly6G antibodies on ice for 60 min, collected and resuspended in PBS. Finally, the cells were examined by flow cytometry (FACS), and the data were analyzed using FACSDiva software (Becton Dickinson, San Jose, CA, USA). Cells were considered to be macrophages on the basis of F4/80^+^ and Ly6G^− ^^42, 43^.

### Statistical analysis

All experiments were repeated at least three times (n ≥ 3). The data for all experiments are presented as the means ± standard deviation (SD), and the results were analyzed using two-tailed Student’s *t*-test to determine significant differences. A value of p < 0.05 was considered statistically significant.

## Electronic supplementary material

Supplementary Information
