# Supplementary material for: Identification and activation of TLR4-mediated signalling pathways by alginate-derived guluronate oligosaccharide in RAW264.7 macrophages
Source: Sci Rep. 2017 May 10;7:1663. doi: 10.1038/s41598-017-01868-0 (PMC5431981; doi:10.1038/s41598-017-01868-0)
Supplement: Supplementary file 1 — Supplementary Information [file 41598_2017_1868_MOESM1_ESM.pdf]

Identification and activation of TLR4-mediated signalling pathways by alginate-derived guluronate oligosaccharide in RAW264.7 macrophages

Weishan Fang [1,*], Decheng Bi [1,*], Ruijin Zheng [2,*], Nan Cai [1], Hong Xu [1], Rui Zhou [1], Jun Lu [1,3], Min Wan [4], Xu Xu [1]

[1] College of Life Sciences and Oceanography, Shenzhen Key Laboratory of Marine Bioresources and Ecology, Shenzhen University, Shenzhen 518060, PR China.

[2] Department of Environmental & Community Medicine, Rutgers University-Robert Wood Johnson Medical School, Piscataway, NJ 08854, USA.

[3] School of Science and School of Interprofessional Health Studies, Faculty of Health and Environmental Sciences, and Institute of Biomedical Technology, Auckland University of Technology, Auckland 1142, New Zealand.

[4] Division of Physiological Chemistry 2, Department of Medical Biochemistry and Biophysics, Karolinska Institute, Stockholm 17177, Sweden.

Correspondence and requests for materials should be addressed to X. X. (email: xuxu@szu.edu.cn)

* These authors contributed equally to this work.

Figure 2C

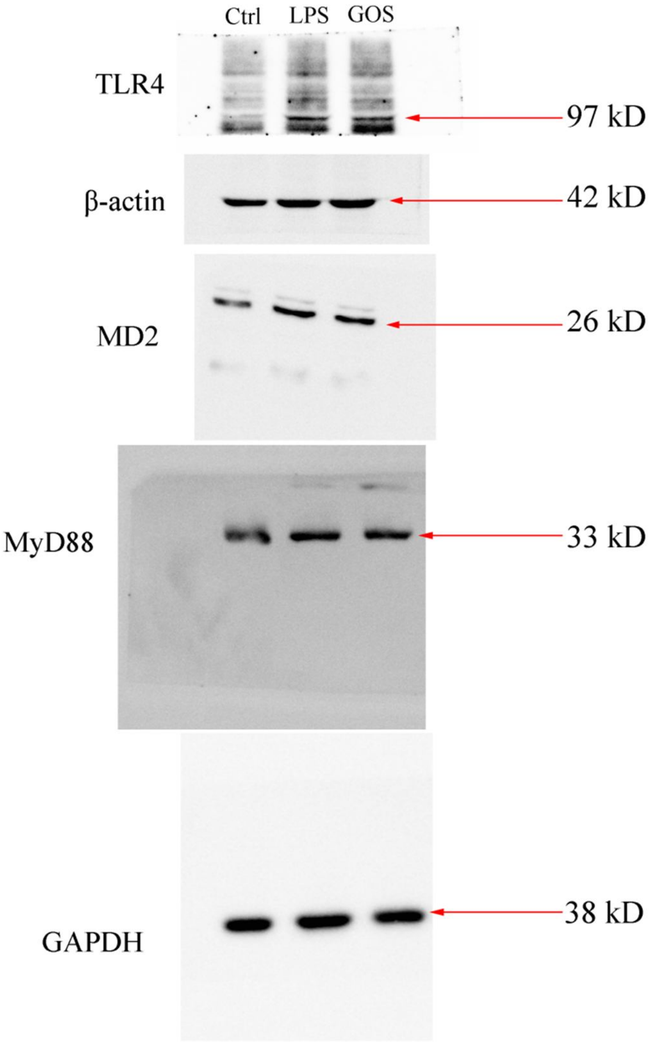
Ctrl
LPS
GOS
TLR4
97 kD
β-actin
42 kD
MD2
26 kD
MyD88
33 kD
GAPDH
38 kD

Figure 2D-E

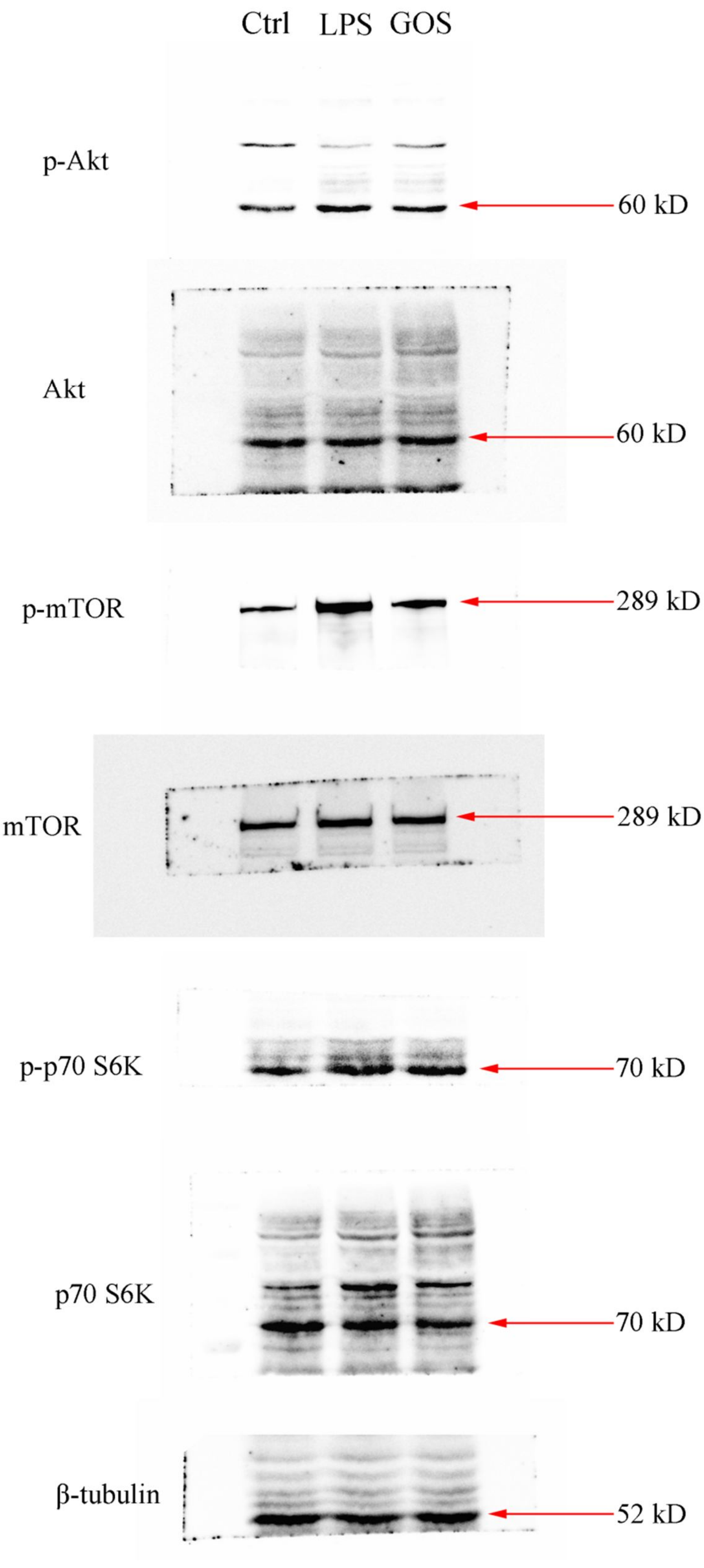
Ctrl
LPS
GOS
p-Akt
60 kD
Akt
60 kD
p-mTOR
289 kD
mTOR
289 kD
p-p70 S6K
70 kD
p70 S6K
70 kD
β-tubulin
52 kD

Figure 3A

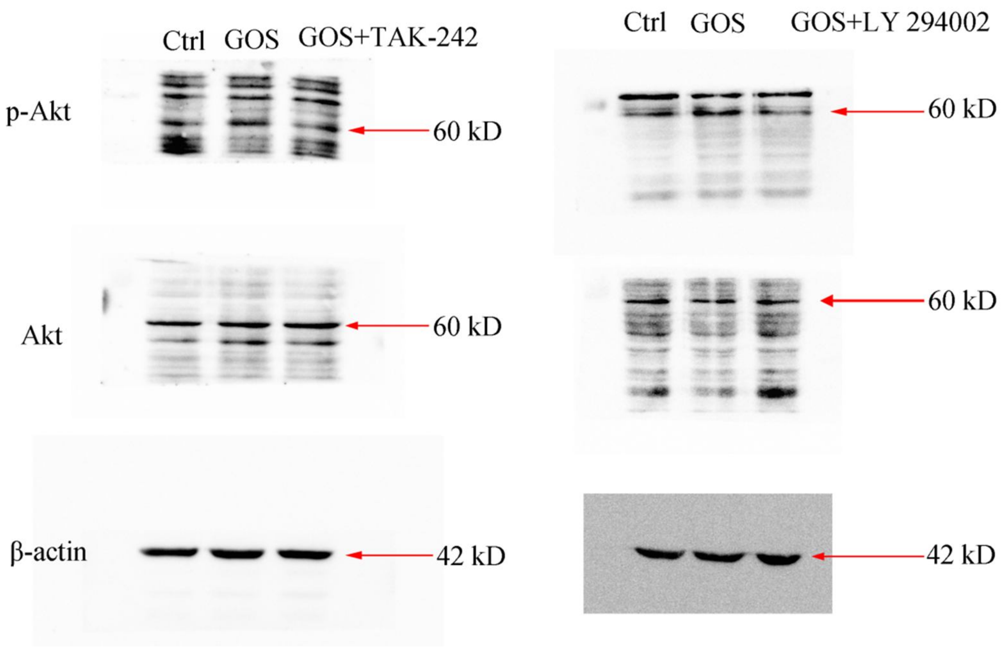
Ctrl GOS GOS+TAK-242
p-Akt
60 kD
Akt
60 kD
β-actin
42 kD
Ctrl GOS GOS+LY 294002
60 kD
60 kD
42 kD

Figure 3B

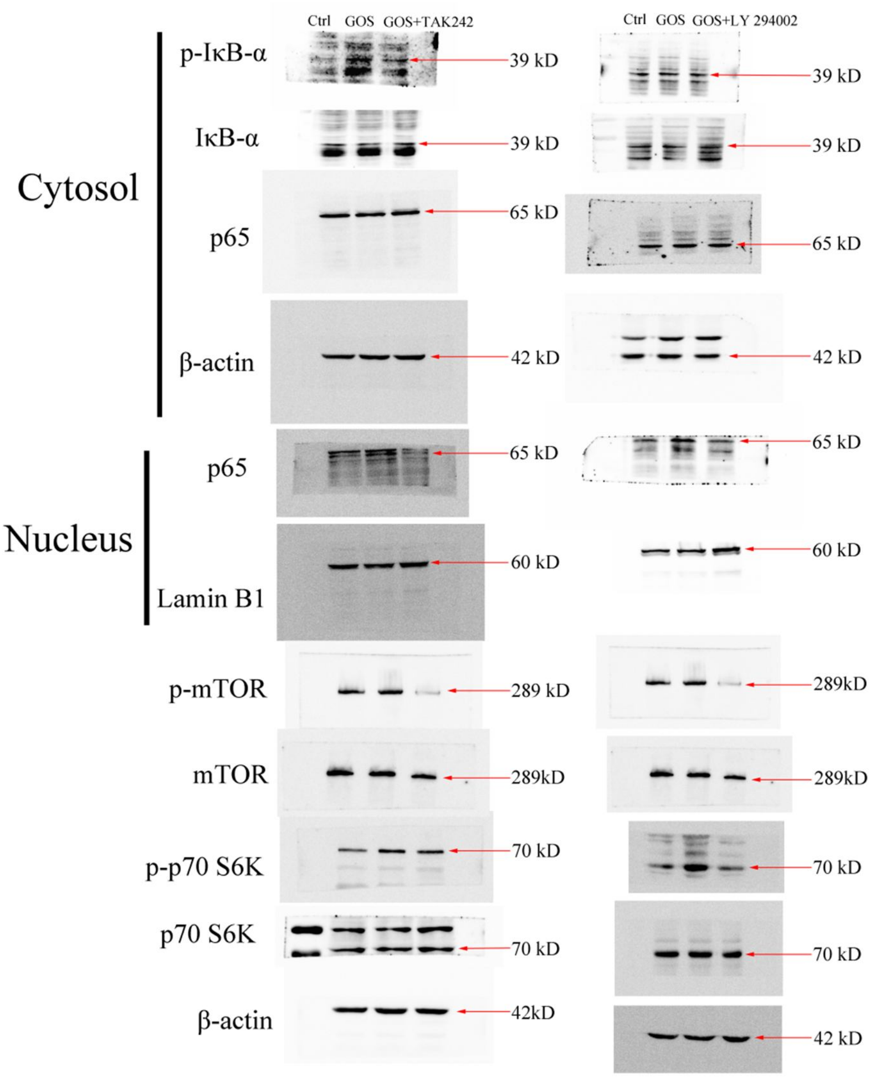
Ctrl GOS GOS+TAK242
Ctrl GOS GOS+LY 294002
Cytosol
p-IκB-α
39 kD
IκB-α
39 kD
p65
65 kD
β-actin
42 kD
Nucleus
p65
65 kD
Lamin B1
60 kD
p-mTOR
289 kD
mTOR
289kD
p-p70 S6K
70 kD
p70 S6K
70 kD
β-actin
42kD
42 kD

Figure 3C

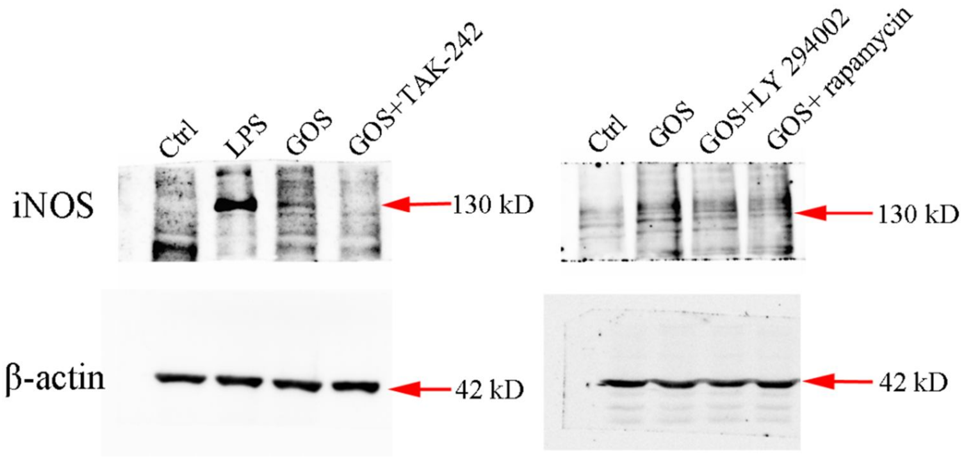
Ctrl
LPS
GOS
GOS+TAK-242
iNOS
130 kD
β-actin
42 kD
Ctrl
GOS
GOS+LY 294002
GOS+ rapamycin
130 kD
42 kD

Figure 4A

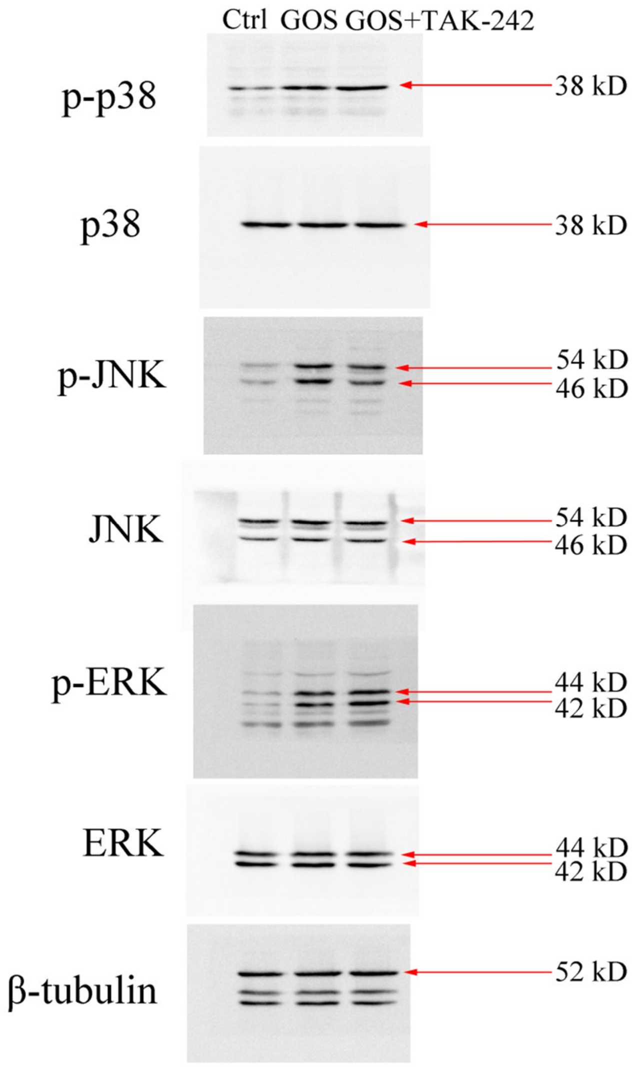
Ctrl GOS GOS+TAK-242
p-p38
38 kD
p38
38 kD
p-JNK
54 kD
46 kD
JNK
54 kD
46 kD
p-ERK
44 kD
42 kD
ERK
44 kD
42 kD
β-tubulin
52 kD

Figure 4B

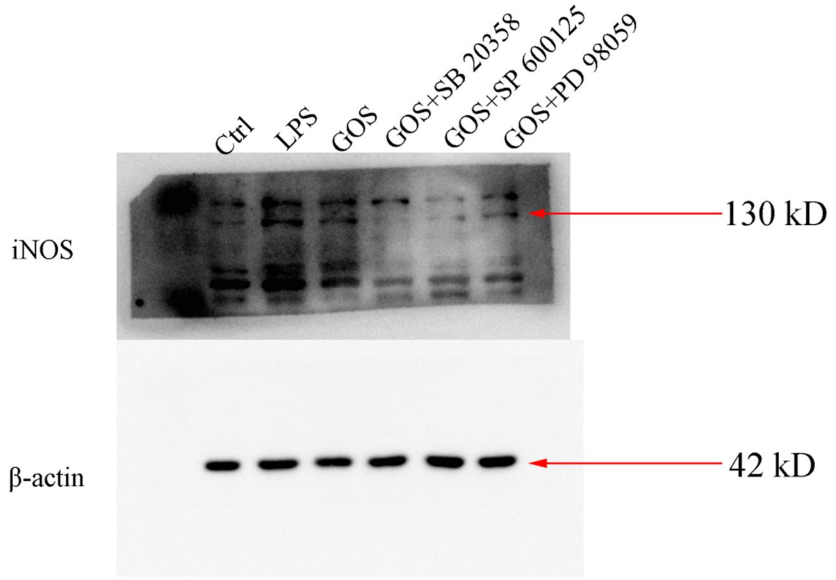

Ctrl
LPS
GOS
GOS+SB 20358
GOS+SP 600125
GOS+PD 98059
iNOS
130 kD
β-actin
42 kD

Figure 5A-F

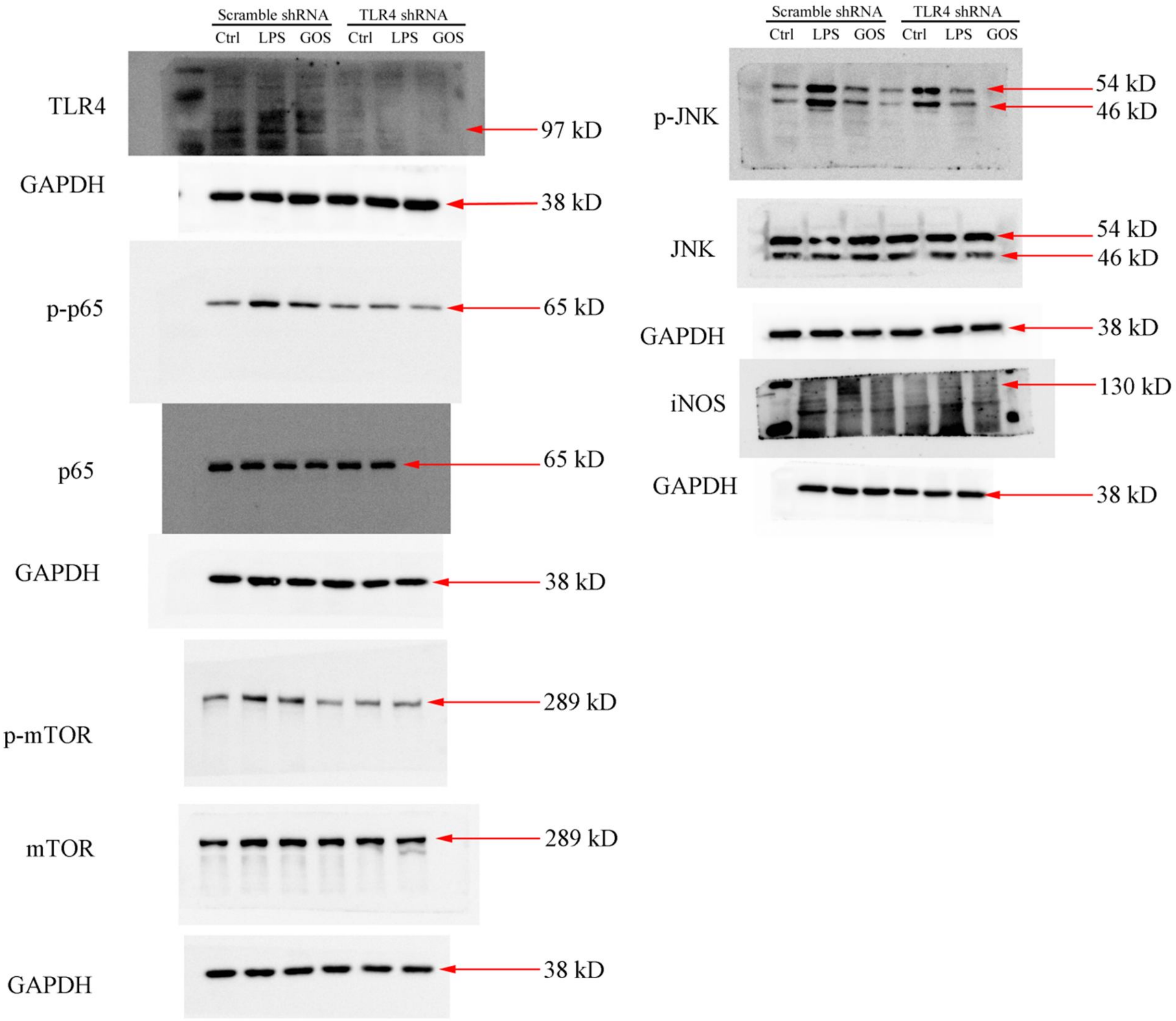

Scramble shRNA
TLR4 shRNA
Ctrl LPS GOS Ctrl LPS GOS
TLR4
97 kD
GAPDH
38 kD
p-p65
65 kD
p65
65 kD
GAPDH
38 kD
p-mTOR
289 kD
mTOR
289 kD
GAPDH
38 kD
Scramble shRNA
TLR4 shRNA
Ctrl LPS GOS Ctrl LPS GOS
p-JNK
54 kD
46 kD
JNK
54 kD
46 kD
GAPDH
38 kD
iNOS
130 kD
GAPDH
38 kD
